# Bone marrow mesenchymal stem cells-derived exosomal lncRNA GAS5 mitigates heart failure by inhibiting UL3/Hippo pathway-mediated ferroptosis

**DOI:** 10.1186/s40001-024-01880-x

**Published:** 2024-05-30

**Authors:** Yu Ren, Xingsheng Zhao

**Affiliations:** 1 Department of Scientific Research, Inner Mongolia People’s Hospital, Hohhot, 010017 China; 2 Department of Cardiology, Inner Mongolia People’s Hospital, No.20 Zhao Wuda Road, Hohhot, 010017 China

**Keywords:** Bone marrow mesenchymal stem cell, Exosome, Heart failure, Ferroptosis, lncRNA GAS5

## Abstract

**Background:**

Exosomes (Exos) are involved in the therapeutic effects of bone marrow mesenchymal stem cells (BMSCs) on heart failure (HF). We investigated the molecular mechanisms underlying the involvement of BMSC-Exos in ferroptosis on HF.

**Methods:**

A rat model of HF and cellular model of hypoxia were established. BMSC-Exos were injected into model rats or co-cultured with model cells. In model rats, the cardiac function (echocardiography), oxidative stress (commercial kits), pathological damage (HE staining), fibrosis (MASSON staining), iron deposition (Prussian blue staining), and cell apoptosis (TUNEL staining) were examined. Viability (cell counting kit-8; CCK-8), cell cycle (flow cytometry), oxidative stress, and Fe^2+^ levels were detected in the model cells. GAS5, UL3, YAP, and TAZ expression were detected using qRT-PCR, western blotting, and immunohistochemistry analyses.

**Results:**

BMSC-Exos restored cardiac function and inhibited oxidative stress, apoptosis, pathological damage, fibrosis, and iron deposition in myocardial tissues of HF rats. In hypoxic cells, BMSC-Exos increased cell viability, decreased the number of G1 phase cells, decreased Fe^2+^ levels, and inhibited oxidative stress. Ferrostatin-1 (a ferroptosis inhibitor) exhibited a synergistic effect with BMSC-Exos. Additionally, GAS5 was upregulated in BMSC-Exos, further upregulating its target UL3 and Hippo pathway effectors (YAP and TAZ). The relieving effects of BMSC-Exos on HF or hypoxia-induced injury were enhanced by GAS5 overexpression, but weakened by UL3 silencing or verteporfin (a YAP inhibitor).

**Conclusions:**

GAS5-harbouring BMSC-Exos inhibited ferroptosis by regulating the UL3/Hippo pathway, contributing to HF remission in vivo and in vitro.

## Background

Heart failure (HF) is a clinical syndrome caused by cardiac overload and injury [1]. HF prevalence is approximately 1–2% globally, depending on the differences in race, region, and definition [2, 3]. Additionally, HF is considerably more prevalent in the elderly, and its incidence continues to rise coupled with a growing and ageing population [4]. Because the oxygen supply fails to meet the requirements of human tissues, HF greatly limits the quality of life and threatens human lives [5]. Bone marrow mesenchymal stem cells (BMSCs)-based therapy is an emerging strategy for HF treatment [6]. Emerging evidences, including clinical trials, have determined that BMSC transplantation is feasible and safe for improving ventricular function in HF [7]. However, the molecular mechanisms underlying the involvement of BMSCs in HF are not yet fully understood.

Exosomes (Exos) are cell-derived nanovesicles involved in intercellular communication and transport various nucleic acids, proteins, lipids, and bioactive substances [8]. Stem cell-derived Exos are critical for paracrine mechanism-based stem cell-based therapies [9]. Exosomal miRNA-30e from BMSCs and exosomal miRNA-1246 from umbilical cord MSCs reportedly ameliorate HF [10, 11]. Long non-coding RNAs (lncRNAs) are also critical regulators of MSC-Exos and participate in the therapeutic mechanisms of MSCs in cardiovascular diseases [12]. Mao et al. [13] report that lncRNA KLF3-AS1 in MSC-Exos attenuates myocardial infarction by inhibiting pyroptosis in cardiomyocytes [13]. Li et al. [49] observe that lncRNA histocompatibility leukocyte antigen complex P5 (HCP5) in BMSC-Exos protects cardiomyocytes against ischemia/reperfusion (IR) injury [14]. However, the lncRNAs involved in the action mechanisms of BMSC-Exos in HF have rarely been reported. Growth-arrest-specific 5 (GAS5) is a specific lncRNA that acts as a tumour suppressor in multiple cancer types [15]. GAS5 also reportedly plays a key regulatory role in the pathogenesis of cardiovascular diseases such as coronary artery disease [16], myocardial infarction [17], coronary atherosclerosis [18], and diabetic cardiomyopathy [19]. Notably, Du et al. [20] reveal that silencing GAS5 protects H9C2 cells against hypoxia-induced injury, probably contributing to the remission of myocardial infarction-induced HF [20]. Patel et al. report that GAS5 is enriched in human adipose stem cell-derived exosomes [21]. Therefore, we speculated that exosomal GAS5 participates in the therapeutic mechanisms of BMSCs in HF.

Since lncRNAs are non-coding RNAs, their functions depend on the relevant target genes [22]. Recently, several downstream target genes of GAS5 have been identified in diverse cardiovascular diseases, including *CALM2* and *PDCD4* in myocardial infarction [17, 23], *TXNIP* in coronary atherosclerosis [18], *CYP11B2* in diabetic cardiomyopathy [24], *P2Y12* in coronary artery disease [25], and *ROCK1* in myocardial ischaemia/reperfusion injury [26]. UL3, also known as RPL3, is a ribosomal protein that belongs to the L3P family. Recent studies on UL3 have mainly focused on cancers, indicating a key regulatory role of UL3 in cytoprotective autophagy [27], cell cycle arrest [28], nucleolar stress [29], and oxidative stress [30]. However, the regulatory role of UL3 in HF and its relevant relationship with GAS5 remains unclear.

Iron is essential for life, but its overload also initiates unique programmed cell death, termed ferroptosis [31]. Ferroptosis is implicated in multiple human diseases such as cancer, renal failure, liver failure, myocardial IR injury, and neurodegenerative diseases [32]. Ferroptosis is associated with iron-induced oxidative damage, thereby contributing to the loss of cardiomyocytes in HF [33]. In addition, Chen et al. [34] determined that toll-like receptor 4 (TLR4) or NADPH oxidase 4 (NOX4) silencing inhibits activated autophagy and ferroptosis in rats with HF, providing potential therapeutic targets for HF [34]. Therefore, ferroptosis inhibition may be beneficial for HF treatment. In this study, we aimed to clarify how BMSCs-Exos treat HF and emphasise the mechanism of BMSCs-Exos involving in the ferroptosis and GAS5/UL3/Hippo pathway. This study revealed the relevant mechanisms of BMSC-Exos in alleviating HF, laying the foundation for the application of BMSCs in the clinical treatment of HF.

## Methods

### Isolation and identification of BMSCs-Exos

Human BMSCs (SNP-H096, SUNNCELL, Wuhan, China) were cultured in Dulbecco’s modified Eagle medium (DMEM) supplemented with 10% foetal bovine serum (FBS) and 100 μg/mL penicillin/streptomycin at 37 °C with 5% CO_2_. Exos were isolated from BMSCs by ultracentrifugation and labelled with PKH67 (green fluorescence). The morphology of the BMSC-Exos was observed under a transmission electron microscope (JEM-1400 Flash, JEOL, Japan), and the particle size was measured using a Flow NanoAnalyzer (NanoFCM, Xiamen, China). In addition, western blotting was performed to detect the expression of surface markers (CD63 and CD81) in the BMSC-Exos (details are described later).

### Establishment of a rat model of HF and treatments

Male Sprague–Dawley (SD) rats (1–3 days old, 180–220 g) purchased from HFK Bioscience (Beijing, China) were acclimatised for a week in the laboratory at 22 ℃ and 50–60% humidity with access to food and water ad libitum. Transverse aortic coarctation (TAC) is commonly used for HF model in previous publications [35, 36], so TAC model was established for the following exploration in our study as previous publications. Specifically, rats were first anaesthetised using pentobarbital sodium (60 mg/kg), and normal breathing was maintained by tracheal cannula (tidal volume = 2–3 ml/100 g; frequency = 60–80 times/minute; respiration ratio = 1:1). Then, the chest of rat was opened, the aorta was separated, and a silk suture was used to ligate the arterial segment between the truncus brachiocephalicus and aortic root. The constriction maintained ~ 70% of the original diameter of the aorta (note: less than 70% of the diameter easily leads to sudden cardiac arrest). The animal experiments were approved by the ethical committee of Xiamen University in accordance with the Guide for the Care and Use of Laboratory Animals (XMULAC20220034-18).

All the rats were divided into following groups: sham, Model + PBS, Model + Exos (BMSC-Exos), Model + ferrostatin-1 (Fer-1, ferroptosis inhibitor), Model + Exos + Fer-1, and Model + Exos + GAS5 (lenti-oe-GAS5), with three replications in each group. Rats that underwent surgery without TAC were assigned to the sham group; the model rats were intra -myocardially injected with 50 μL PBS; the rats in Model + Exos group were injected 50 μL PBS containing BMSCs-Exo; the rats in Model + Fer-1 group were injected 50 μL PBS containing Fer-1 (2 mg/kg, ABclonal RM02804, Wuhan, China); the rats in Model + Exos + Fer-1 group were injected 50 μL PBS containing BMSCs-Exo and Fer-1. The concentration of Fer-1 referenced a publication [37]. After three weeks, the animals were killed for the subsequent experiments.

### Evaluation of cardiac function

After modelling for 21 days, cardiac function parameters, including left ventricular end-diastolic diameter (LVEDD), end-systolic diameter (LVESD), ejection fraction (EF), and fractional shortening (FS), were measured by echocardiography using an animal ultrasound imaging system (VisualSonics Vevo 2100, Toronto, Canada).

### Histopathological evaluation

After measuring cardiac function, the rats were anaesthetised and killed by cervical dislocation. Myocardial tissues were resected, fixed in 10% formaldehyde, embedded in paraffin, and sliced into 5 μm sections. After dewaxing and rehydration, the sections were stained with haematoxylin–eosin (HE) (Beyotime, Beijing, China) to determine pathological damage, MASSON (Haematoxylin, Masson's ponceau acid fuchsin solution, and aniline blue; Solarbio, Beijing, China) to determine fibrosis, Prussian blue (Solarbio) to determine iron deposition, or terminal deoxynucleotidyl transferase dUTP nick end labelling (TUNEL; Beyotime) to determine apoptosis. A part of each section was used to detect UL3 expression by immunohistochemistry (IHC). Stained sections were observed under a microscope (Olympus, Tokyo, Japan).

### Establishment of a hypoxia model in myocardial cells and treatments

H9C2 cells, a rat myocardial cell line (American Type Culture Collection, Manassas, VA, USA), is used for HF cell model construction [38, 39], and were cultured in DMEM supplemented with 10% FBS and 100 μg/mL penicillin/streptomycin at 37 °C with 5% CO_2_. The hypoxia model was established by culturing the cells for 6 h in an atmospheric environment of 95% N_2_ and 5% CO_2_ (model cells). H9C2 cells cultured under normal oxygen were used as the normal control (NC) group. The model cells were transfected with different agents according to the following groups: Model + PBS, Model + Exos (BMSCs-Exos), Model + Fer-1, Model + Exos + Fer-1, Model + Exos + GAS5 (lenti-oe-GAS5), Model + Exos + oe-NC (lenti-oe-NC), Model + Exos + Ver (Verteporfin; Adooq, Nanjing, China), Model + Exos + shNC, Model + Exos + shUL3, Model + Exos + shUL3 + oe-NC, and Model + Exos + shUL3 + GAS5. Overexpression vectors carrying GAS5 (oe-GAS5), empty overexpression vector (oe-NC), shRNA-UL3 (sh-UL3), and shRNA-NC (sh-NC) were packaged in lentivirus (GenePharma, Shanghai, China) and transfected into cells using Highgene transfection reagent (ABclonal). The concentration of Fer-1 used in vitro is 1 mmol/L. There were three replications in each group.

### Western blotting

Total proteins were extracted from BMSC-Exos, myocardial tissues, and myocardial cells by lysis in RIPA buffer (Beyotime). The protein concentration was measured using a BCA kit (Beyotime), and the proteins were separated by 10% SDS polyacrylamide gel electrophoresis and transferred onto PVDF membranes (Beyotime). Subsequently, the membranes were blocked with 5% non-fat milk and incubated with specific primary antibodies (anti-CD63, -CD81, -UL3, -YAP, -TAZ, -ACSL4, -GPX4, and -GAPDH; 1:2,000, Abcam, Cambridge, UK) overnight at 4 °C, followed by incubation with HRP-conjugated secondary antibody (goat anti-rabbit IgG, 1:5000, Abcam) for 1 h at 25 °C. Protein bands were visualised using an ECL reagent (Thermo Fisher Scientific, Waltham, MA, USA) and observed using a gel imaging system (Tanon 3500, China).

### Quantitative real time-polymerase chain reaction (qRT-PCR)

Total RNAs were extracted from BMSC-Exos, myocardial tissues, or myocardial cells using TRIzol reagent (Thermo Fisher Scientific) and immediately reverse-transcribed into cDNAs using a cDNA Synthesis kit (Tiangen, Beijing, China). qRT-PCR was performed on an Mx3000P instrument (Stratagene, Carlsbad, CA, USA) at 95 °C for 3 min, followed by 40 cycles at 95 °C for 12 s and 62 °C for 40 s. Relative expression levels were quantified according to the 2^−∆∆Ct^ method using *GAPDH* as an internal control. The primers used in qRT-PCR included *GAS5* (human)-F, 5′-TTC TGC GTT AGG AAG CCT GG-3′, *GAS5* (human)-R, 5′-CAA GCC GAC TCT CCA TAC CC-3′; *GAPDH* (human)-F, 5′-TGT GGG CAT CAA TGG ATT TGG-3′, *GAPDH* (human)-R, 5′-ACA CCA TGT ATT CCG GGT CAA T-3′; *GAS5* (rat)-F, 5′-AAC TGA CTT TAT GCT TGC CC-3′, *GAS5* (rat)-R, 5′-CCA TCT TCC ACC TGT AGG GT-3′; *UL3* (rat)-F, 5′-GGT GAC CAG TCG TTG GCA TA-3′, *UL3* (rat)-R, 5′-TGC GAT CTT TCT TGA GCG GT-3′; *GAPDH* (rat)-F, 5′-GCG AGA TCC CGC TAA CAT CA-3′, *GAPDH* (rat)-R, 5′-CTC GTG GTT CAC ACC CAT CA-3′.

### Measurement of oxidative stress parameters, Fe^2+^, and ATP levels

In﻿ the serum and supernatants of model rats and cells, respectively, the levels of oxidative stress parameters (malondialdehyde [MDA], reactive oxygen species [ROS], superoxide dismutase [SOD], and glutathione [GSH]), Fe^2+^, and ATP levels were measured using commercial kits (MDA/ROS/SOD, Solarbio; GSH, Elabscience, Wuhan, China; Fe^2+^, BioVision, Milpitas, CA, USA; ATP, Solarbio) as per the manufacturer’s instructions.

### Cell counting kit-8 (CCK-8) assay

H9C2 cell viability was determined using the CCK-8 kit (Beyotime). Briefly, H9C2 cells were seeded in 96-well plates and co-cultured with BMSC-Exos (with or without other treatments) for 12 and 24 h, respectively. After incubation with CCK-8 for 2 h, the optical density at 450 nm was measured using a microplate reader (DR-3518G, Hiwell Diatek, Wuxi, China).

### Cell cycle assay

The cell cycle of H9C2 cells was detected by flow cytometry. Briefly, the cells were digested with trypsin and fixed in 70% ethanol for 6 h at 4 °C. After incubation with RNase A for 30 min at 37 °C, cells were stained with PI (Beyotime) for 30 min in the dark. Cells in the G1 phase were monitored using a flow cytometer (CytoFLEX S, Beckman), and the relative percentage was analysed using Cell Quest software (BD Biosciences, NJ, USA).

### RNA immunoprecipitation (RIP) assay

The target relationship between *GAS5* and *UL3* was identified using RIP assay. Briefly, cells were lysed in RIPA buffer and incubated with beads conjugated with anti-Ago2 or IgG (Geneseed, Guangzhou, China) for 12 h at 4 °C. After centrifugation and 30 min of incubation with proteinase K at 55 °C, the precipitates were collected, and immunoprecipitated RNAs were extracted using TRIzol reagent (Thermo Fisher Scientific). As mentioned earlier, the relative expression of *GAS5* and *UL3* was detected by qRT-PCR.

### *Fluorescence *in situ* hybridisation (FISH)*

The subcellular colocalisation of GAS5 and UL3, as well as UL3 and YAP was detected using a FISH kit (RiboBio, Guangzhou, China). Briefly, cells were fixed in 4% paraformaldehyde for 15 min, permeabilised with 0.1% Triton X-100 for 15 min, soaked in 2 × SSC solution for 30 min, and dehydrated in graded ethanol. After hybridisation with a 1 μg/mL probe for 12 h at 37 °C, the samples were washed in 0.4 × SSC solution containing 0.3% Triton X-100 for 2 min at 65 °C and then in 2 × SSC solution containing 0.1% Triton X-100 for another 2 min at 25 °C. The cells were finally stained with DAPI for 5 min in the dark and observed under a microscope (Olympus).

### Statistical analysis

Data are presented as mean ± standard deviation and were statistically analysed using GraphPad Prism 7.0 (GraphPad, San Diego, CA, USA). Comparisons between two and among multiple groups were analysed by t-test and one-way ANOVA followed by Tukey’s test, respectively. Statistical significance was set at *P* < 0.05.

## Results

### GAS5 is upregulated in BMSCs-Exos

**Fig. 1:**
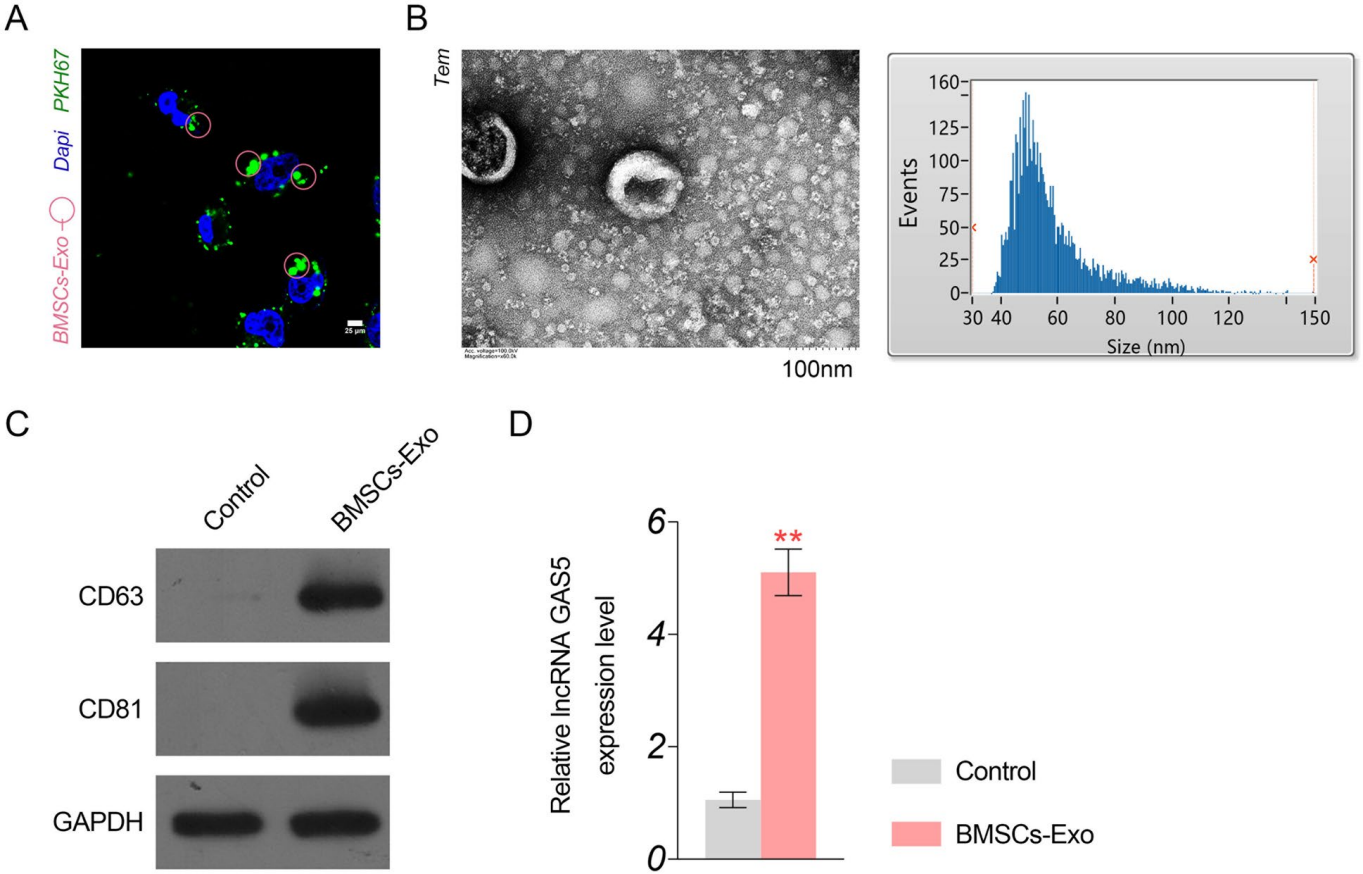
Characteristics of Exos isolated from BMSCs. **A** The green fluorescence of PKH67 indicates presence of BMSCs-Exo under a microscope (scale bar = 25 µm). **B** The morphology of BMSCs-Exos under TEM (scale bar = 100 nm); BMSCs-Exo is a saucer-like, homogeneous, tiny vesicle with a membrane-like structure; the particle size range is 36–140 nm, with an average particle size of 53.25 nm. **C** The protein expression of CD63 and CD81 in BMSCs-Exos detected by western blotting; **D** GAS5 expression in BMSCs-Exos detected by qRT-PCR. ^**^P < 0.01 vs. control

Exos with evident green fluorescence of PKH67 were isolated from the BMSCs (Fig. 1A). Under TEM, BMSC-Exos presented tiny vesicles with membrane-like structures (particle size range, 36–140 nm; average particle size: 53.25 nm) (Fig. 1B). Western blotting subsequently determined that BMSC-Exos were positive for CD63 and CD81 (Exos markers) (Fig. 1C). The above evidences demonstrated the success of BMSC-Exos extraction. Additionally, q-PCR analysis indicated a significantly higher expression of GAS5 in BMSC-Exos than in controls (*P* < 0.01, Fig. 1D).

### BMSCs-Exos alleviate HF in rats by inhibiting ferroptosis

**Fig. 2:**
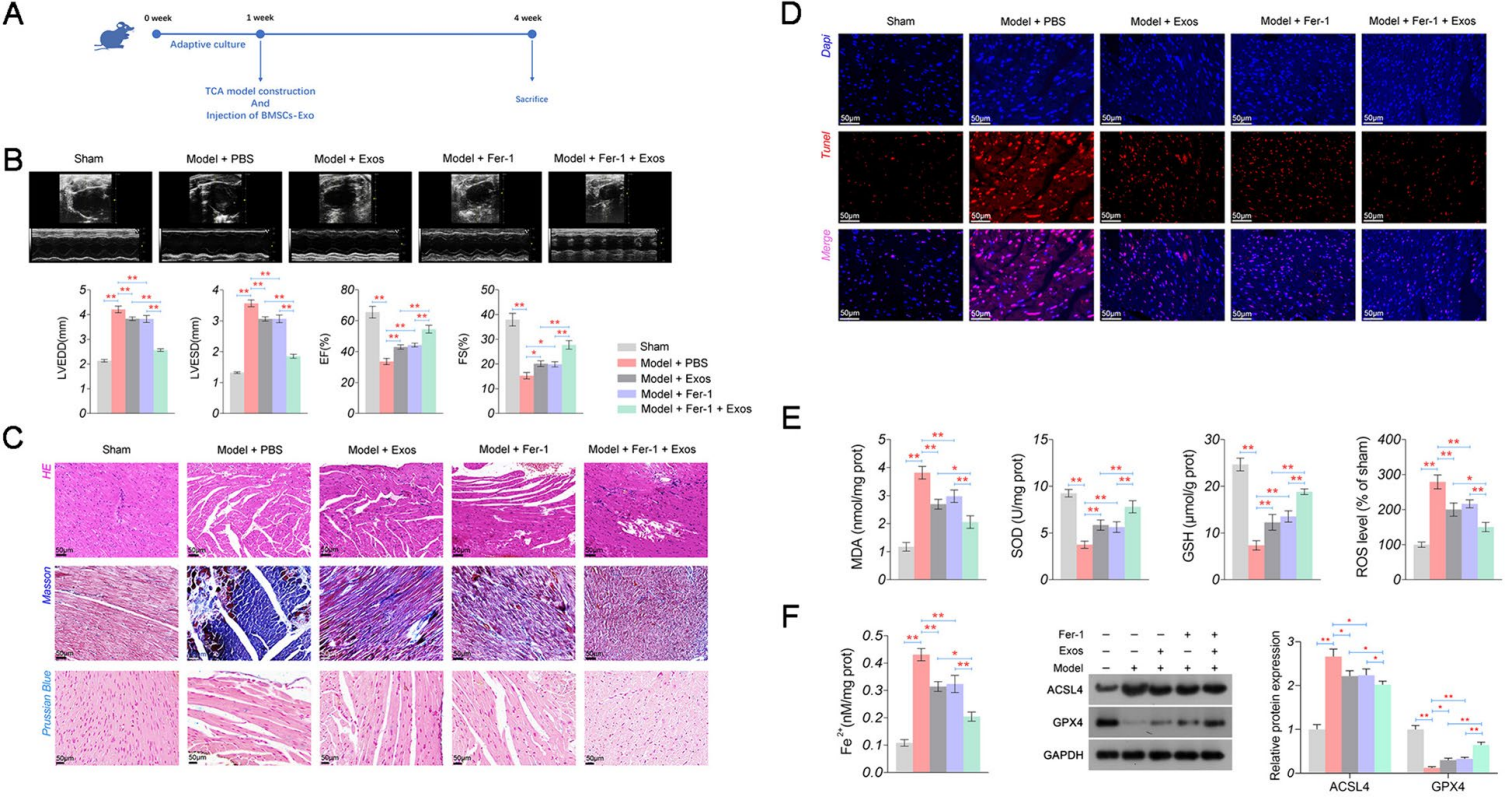
Effects of BMSCs-Exos and ferrostatin-1 on cardiac function, oxidative stress, and ferroptosis in HF rats. **A** The treatment process of the TAC-induced HF rat model. **B** The levels of cardiac function parameters (LVEDD, LVESD, EF, and FS). **C** Pathological damage of myocardial tissues detected by HE staining (scale bar = 50 µm); myocardial fibrosis detected by Masson staining (scale bar = 50 µm); iron deposition detected by Prussian Blue staining (scale bar = 20 µm). **D** Apoptosis of myocardial cells was detected by TUNEL staining (scale bar = 50 µm). **E** The levels of oxidative stress parameters (MDA, SOD, GSH, and ROS). **F** The levels of Fe^2+^ and the protein expression of ACSL4 and GPX4, as ferroptosis parameters. ^*^P < 0.05, ^**^P < 0.01

To further investigate the role of BMSCs-Exos in animals, a TAC-induced HF rat model was established (Fig. 2A). Myocardial function assay indicated that the HF rat model was successfully established, presenting as increased LVEDD and LVESD and decreased EF and FS (*P* < 0.01, Fig. 2B). HE staining showed a disordered arrangement of myocardial fibres and a widened gap of myocardial cells in the model rats (Fig. 2C). The model rats also exhibited myocardial fibrosis and iron deposition in Masson staining and Prussian Blue staining, respectively (Fig. 2C). TUNEL staining suggested enhanced apoptosis of myocardial cells in model rats (Fig. 2D).

Oxidative stress is an important pathophysiological pathway in the development and progression of HF [40] and mediates ferroptosis [41]. So we investigated the changes of oxidative stress-related indicators. The results indicated significantly higher MDA and ROS levels, and lower SOD and GSH levels in the model group than in the sham group (*P* < 0.01, Fig. 2E). Concurrent with these alterations, Fe^2+^ levels and ACSL4 expression, as pivotal indicators of ferroptosis, were markedly augmented in the model group, whereas GPX4 expression was notably downregulated (*P* < 0.01, Fig. 2F).

**Fig. 3:**
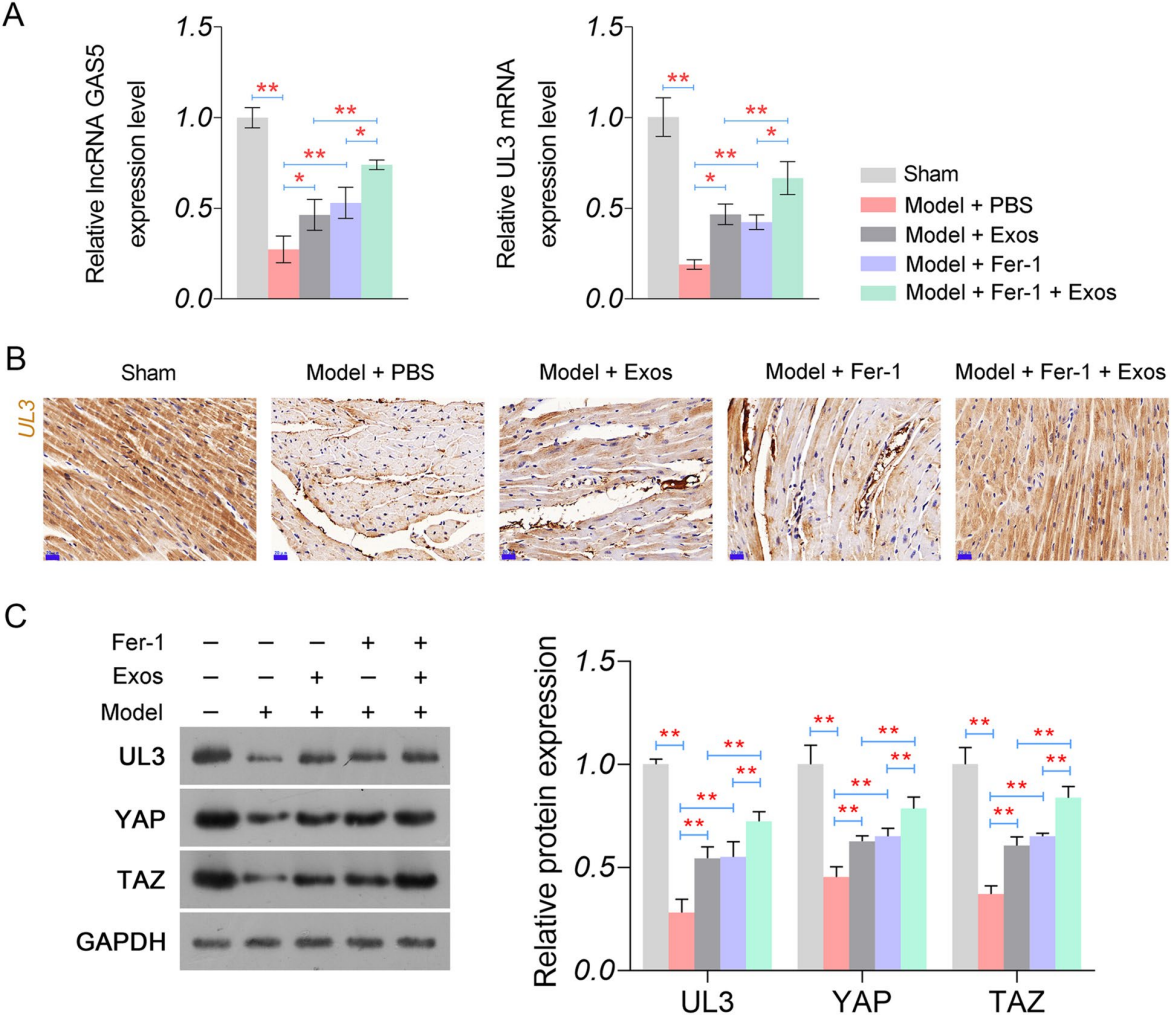
Effects of BMSCs-Exos and ferrostatin-1 on the expression of GAS5 and UL3 in myocardial tissues of HF rats. **A** Expression of *GAS5* and *UL3* detected by qRT-PCR. **B** Protein expression of UL3 was detected by IHC (scale bar = 20 µm). **C** Protein expression of UL3, YAP, and TAZ was detected by western blotting. ^*^P < 0.05; ^**^P < 0.01

After BMSC-Exos injection, cardiac function was partially recovered in the model rats, as evidenced by decreased LVEDD and LVESD and increased EF and FS (*P* < 0.05, Fig. 2B, C). BMSC-Exos injection also alleviated cell apoptosis (Fig. 2D), oxidative stress (*P* < 0.01, Fig. 2E), and ferroptosis (*P* < 0.05, Fig. 2F) in the myocardial tissues of the model rats. Furthermore, Fer-1, an inhibitor of ferroptosis, demonstrated consistent results with those of BMSC-Exos in model rats (Fig. 2B–F). Fer-1 combined with BMSC-Exos also exhibited more potent protective effects on myocardial tissues in HF rats than BMSC-Exos alone (Fig. 2B–F).

### BMSCs-Exos upregulate GAS5 and UL3 in HF rats

The effects of BMSC-Exos on GAS5 and UL3 expression were evaluated in the myocardial tissues of model rats. The expression of GAS5 was significantly lower in model rats than in sham rats (*P* < 0.01, Fig. 3A). Similar to GAS5, the mRNA and protein expression of UL3 were significantly decreased in the model group compared to that in the sham group (*P* < 0.01, Fig. 3A–C). Concurrently, the protein levels of YAP and TAZ, key effectors of the Hippo pathway, were also significantly decreased in the model group (*P* < 0.01, Fig. 3C). Notably, the downregulation of GAS5, UL3, YAP, and TAZ in the model rats was reversed by either BMSC-Exos or Fer-1 treatment (*P* < 0.05, Fig. 3A–C). BMSC-Exos also enhanced the fer-1-mediated upregulation of GAS5, UL3, YAP, and TAZ in model rats (*P* < 0.05, Fig. 3A–C).

### BMSCs-Exos alleviate hypoxia-induced myocardial injury by inhibiting ferroptosis

**Fig. 4:**
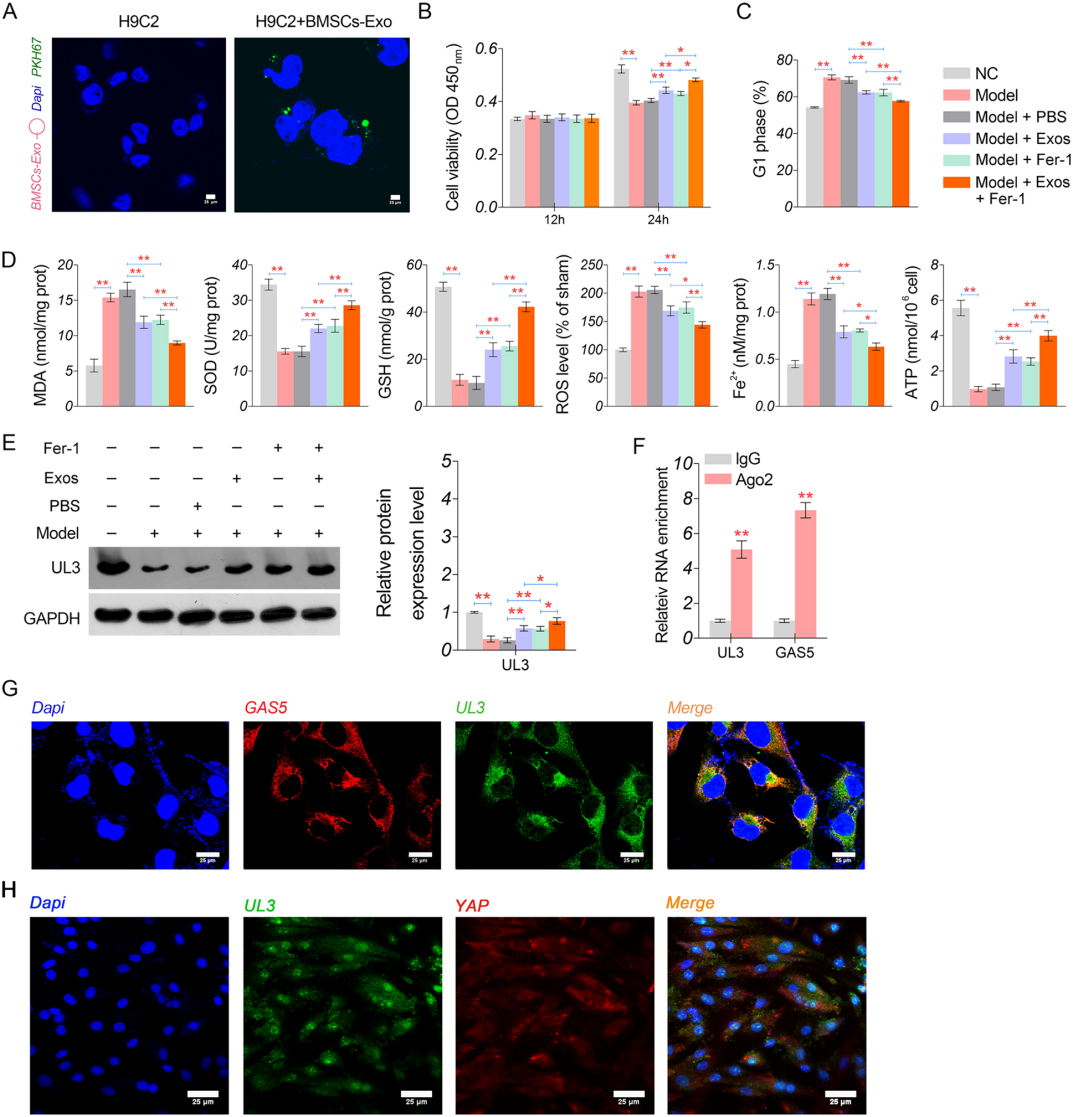
Effects of BMSCs-Exos and ferrostatin-1 on hypoxia-induced injury in H9C2 cells. **A** The fluorescence of PKH67 in H9C2 cells co-cultured with BMSCs-Exos (scale bar = 25 µm). The green fluorescence indicates present of BMSCs-Exo. **B** Cell viability measured by CCK-8 assay. **C** Cells in G1 phase were measured by flow cytometry. **D** The levels of oxidative stress parameters (MDA, SOD, GSH, and ROS), Fe^2+^, and ATP. **E** Protein expression of UL3 detected by western blotting. **F** The target relationship between *GAS5* and *UL3* identified by RIP assay. **G** The subcellular localisation of GAS5 and UL3 detected by FISH assay (scale bar = 25 µm). **H** The subcellular localisation of UL3 and YAP detected by FISH assay (scale bar = 25 µm). ^*^P < 0.05; ^**^P < 0.01

H9C2 cells were co-incubated with BMSC-Exos to assess the impact on hypoxia-induced injury in vitro. Post 24 h co-incubation, pronounced green fluorescence from PKH67 was discerned in H9C2 cells (Fig. 4A), indicating stable growth of BMSC-Exos after entering cardiomyocytes. Relative to normoxic controls, hypoxia-exposed H9C2 cells (referred to as model cells) displayed decreased cell viability (24 h), elevated proportion of cells in the G1 phase, increased oxidative stress (manifested by increased MDA and ROS, along with decreased SOD and GSH), elevated Fe^2+^ levels, and depleted ATP levels (*P* < 0.01, Fig. 4B-D). These hypoxia-induced perturbations in the model cells were partly mitigated by either BMSC-Exos or Fer-1 treatment (*P* < 0.01). Combined treatment with BMSC-Exos and fer-1 exhibited a synergistic effect in ameliorating hypoxia-induced myocardial injury in H9C2 cells (*P* < 0.05, Fig. 4B–D). Moreover, both BMSC-Exos and fer-1 acted synergistically to counteract the hypoxia-induced downregulation of UL3 in the model cells (*P* < 0.05, Fig. 4E). The RIP assay substantiated that GAS5 co-immunoprecipitated with UL3 in H9C2 cells, thus suggesting a potential regulatory interaction between GAS5 and UL3 (*P* < 0.01, Fig. 4F). Intriguingly, FISH revealed a cytoplasmic colocalisation of GAS5 and UL3, as well as UL3 and YAP (Fig. 4G, H).

### GAS5 overexpression enhances the BMSCs-Exos-mediated alleviation of HF in rats

**Fig. 5:**
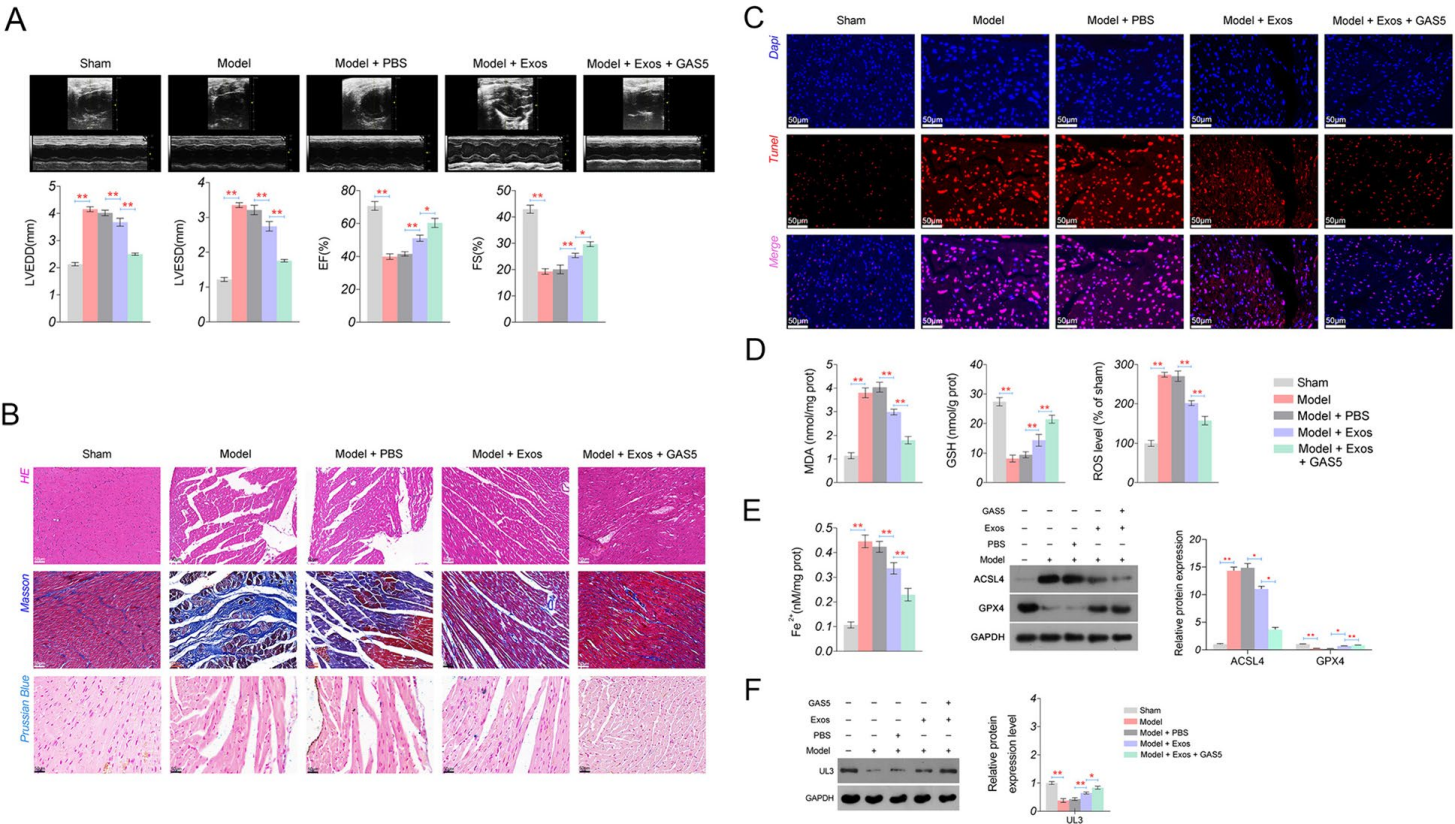
Regulatory effects of GAS5 on cardiac function, oxidative stress, and ferroptosis in HF rats injected with BMSCs-Exos. **A** The levels of cardiac function parameters (LVEDD, LVESD, EF, and FS). **B** Pathological damage of myocardial tissues detected by HE staining (scale bar = 50 µm); myocardial fibrosis detected by Masson staining (scale bar = 50 µm); iron deposition detected by Prussian Blue staining (scale bar = 20 µm). **C** Apoptosis of myocardial cells detected by TUNEL staining (scale bar = 50 µm). **D** The levels of oxidative stress parameters (MDA, GSH, and ROS). **E** The levels of ferroptosis parameters (Fe^2+^, ACSL4, and GPX4). **F** Protein expression of UL3 detected by western blotting. ^*^P < 0.05, ^**^P < 0.01

The mechanisms of action for BMSC-Exos, involving GAS5, were examined in rat models. The TAC-induced HF rat model was successfully established, presented as increased LVEDD and LVESD and decreased EF and FS (*P* < 0.01, Fig. 5A). The overexpression of GAS5 notably amplified the beneficial effects of BMSC-Exos on cardiac function improvement (exhibited through decreased LVEDD and LVESD, and increased EF and FS) (Fig. 5A). Overexpression of GAS5 also enhanced the suppressive effects of BMSC-Exos on pathological damage, fibrosis, ferroptosis, and cell apoptosis in the myocardial tissues of the rat models (Fig. 7B, C). Moreover, overexpression of GAS5 also increased the inhibition of oxidative stress (evidenced by decreased MDA and ROS, and increased GSH) and ferroptosis (characterised by decreased Fe2 + and ACSL4 levels, and increased GPX4 levels) in the rat models (Fig. 5D, E, *P* < 0.05). Furthermore, western blot analysis disclosed that the BMSC-Exos-induced upregulation of UL3 in rat models were further reinforced by GAS5 overexpression (*P* < 0.05, Fig. 5E).

### GAS5 overexpression enhances the BMSCs-Exos-mediated alleviation of hypoxia-induced myocardial injury in vitro

**Fig. 6:**
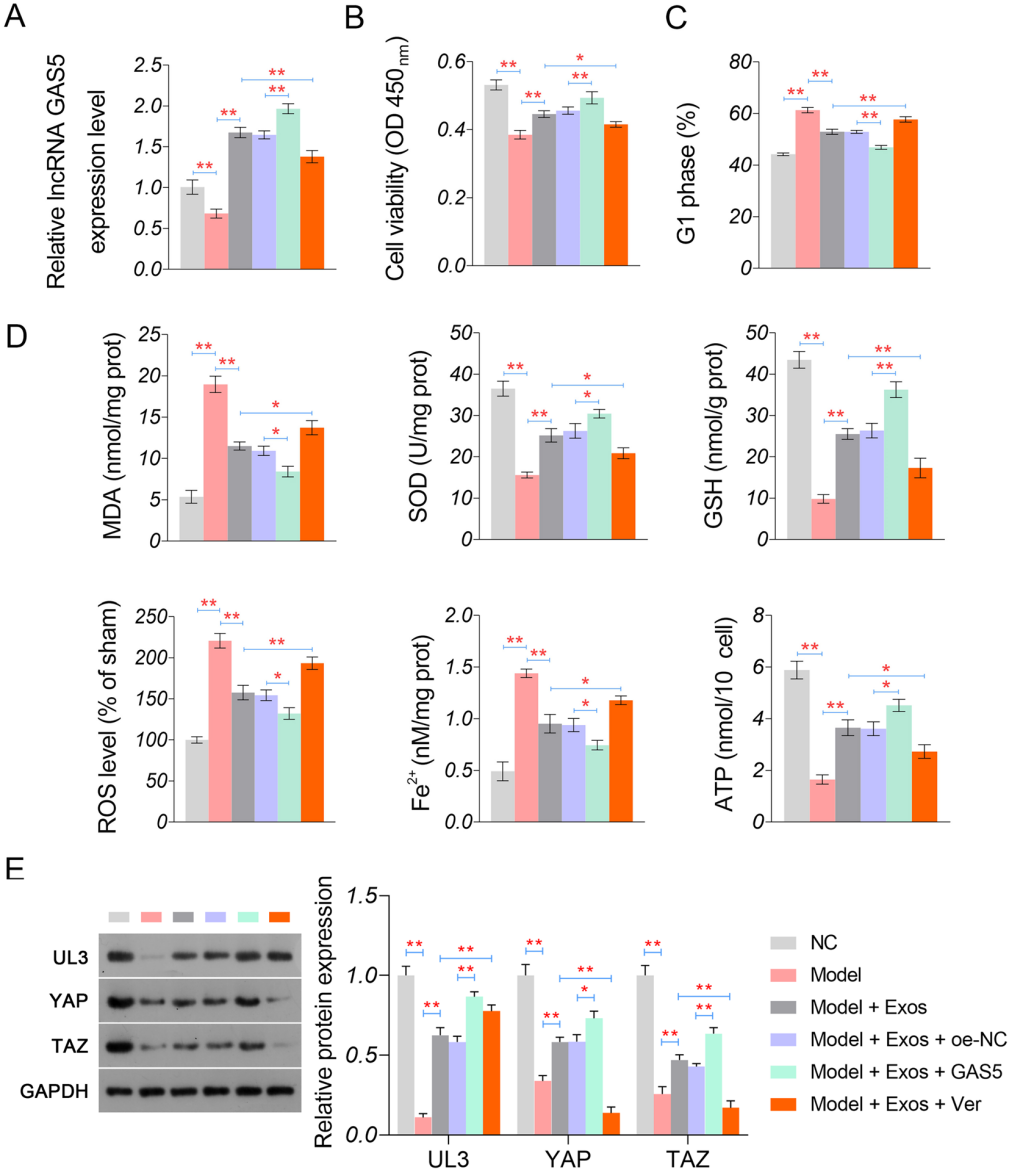
Regulatory effects of GAS5 on hypoxia-induced injury in myocardial cells. **A** The expression of GAS5 detected by qRT-PCR. **B** Cell viability measured by CCK-8 assay. **C** Cells in the G1 phase were measured by flow cytometry. **D** The levels of oxidative stress parameters (MDA, SOD, GSH, and ROS), Fe^2+^, and ATP. E Protein expression of UL3, YAP, and TAZ detected by western blotting. ^*^P < 0.05; ^**^P < 0.01

The action mechanisms of BMSC-Exos involving GAS5 were further analysed in hypoxia-induced myocardial cells (model cells). As shown in Fig. 6A, the transfection of lenti-oe-GAS5 significantly upregulated GAS5 in model cells co-cultured with BMSC-Exos (*P* < 0.01). The subsequent functional experiments demonstrated that GAS5 overexpression enhanced the effects of BMSC-Exos on increasing cell viability and decreasing G1 phase cells (*P* < 0.01, Fig. 6B, C). GAS5 overexpression also strengthened the effects of BMSC-Exos on increasing SOD, GSH, and ATP levels and decreasing MDA, ROS, and Fe^2+^ levels in the model cells (*P* < 0.05, Fig. 6D). Additionally, BMSC-Exos-induced upregulation of UL3, YAP, and TAZ in the model cells was further promoted by GAS5 overexpression (*P* < 0.01, Fig. 6E). In contrast with GAS5, verteporfin, a YAP inhibitor, weakened the effects of BMSC-Exos on upregulating UL3, YAP, and TAZ, as well as inhibiting hypoxia-induced injury in the model cells (*P* < 0.05, Fig. 6B–E).

### *UL3 silencing weakens the BMSCs-Exos-mediated alleviation of hypoxia-induced myocardial injury *in vitro

**Fig. 7:**
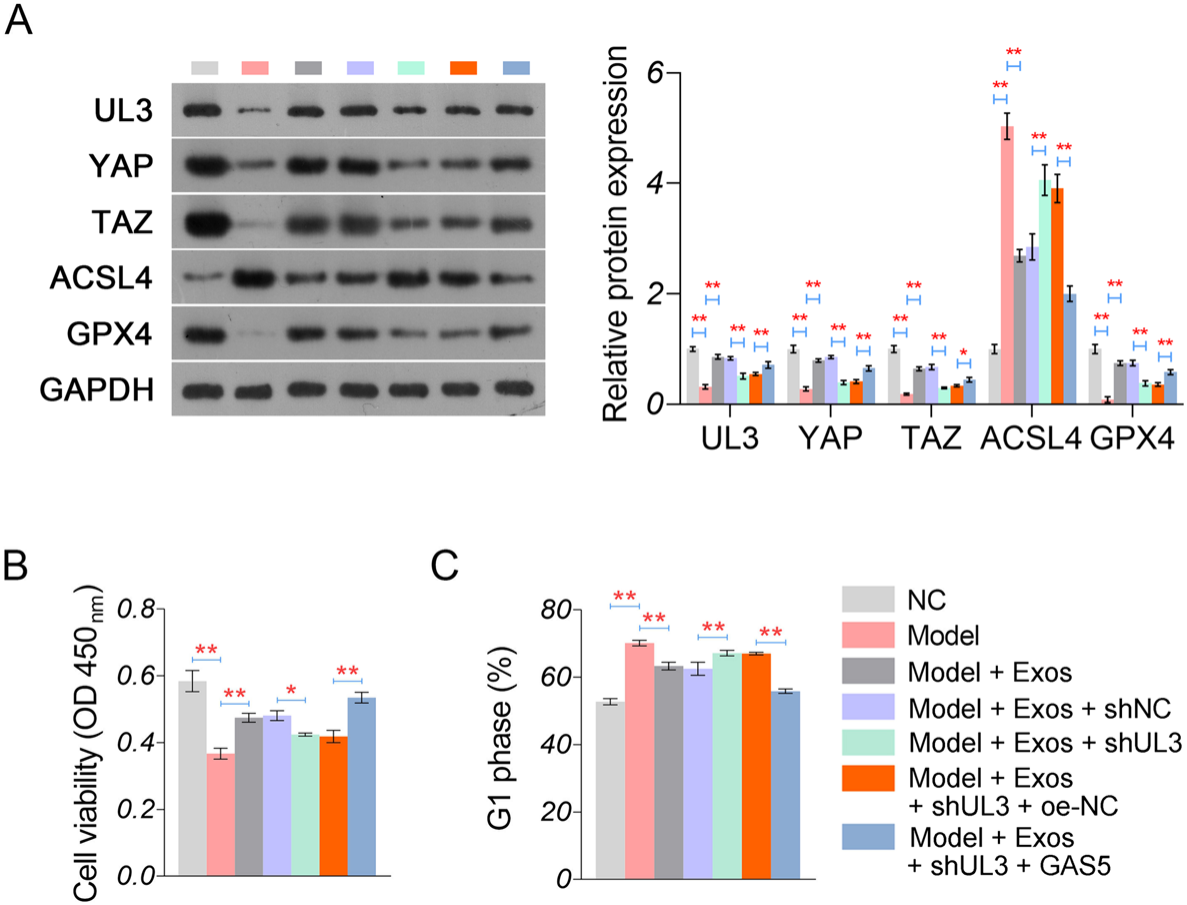
Regulatory effects of UL3 on hypoxia-induced injury in myocardial cells. **A** Protein expression of UL3, YAP, TAZ, ACSL4, and GPX5 detected by western blotting. **B** Cell viability measured by CCK-8 assay. **C** Cells in the G1 phase were measured by flow cytometry. ^*^P < 0.05; ^**^P < 0.01

To determine the action mechanism of BMSC-Exos involving GAS5/UL3, UL3 was silenced in hypoxia-treated H9C2 cells (model cells). Western blotting verified that the transfection of sh-UL3 downregulated UL3, YAP, and TAZ in the model cells co-cultured with BMSC-Exos (*P* < 0.01, Fig. 7A). Additionally, sh-UL3 elevated the ACSL4 expression and decreased the GPX4 expression in the model cells co-cultured with BMSC-Exos (*P* < 0.01, Fig. 7A). Also, the effects of BMSC-Exos on increasing cell viability and decreasing G1 phase cells were weakened by UL3 silencing in model cells (*P* < 0.05, Fig. 7B, C). Furthermore, GAS5 overexpression further restored UL3 expression in sh-UL3-transfected model cells (*P* < 0.01), and the protective effects of BMSC-Exos on hypoxia-induced myocardial injury (*P* < 0.01, Fig. 7A–C).

## Discussion

HF is a public health problem characterised by an inadequate blood supply from the heart to the body [42]. Despite advances in medical and surgical treatments for HF, the mortality associated with ischaemic-origin disorders remains high [7]. BMSCs-based therapy is a promising strategy for HF treatment, in which Exo acts as a critical contributor [6, 9]. In this study, the action mechanisms of BMSC-Exos were evaluated in an HF rat model and a cellular hypoxia model. The results demonstrated that BMSC-Exos with upregulated lncRNAs GAS5 expression relieved HF by inhibiting ferroptosis. Moreover, the GAS5-mediated UL3/Hippo pathway is the underlying mechanism responsible for the therapeutic potential of BMSC-Exos in HF.

lncRNAs play essential regulatory roles in almost all biological processes involved in the pathogenesis of various human diseases, including HF [43]. Many lncRNAs have been identified as potential therapeutic targets for HF, including *CARL*, *APF*, *NRF*, *MHRT*, *H19*, *CHRF*, *CHAST*, and *ROR* [44]. Notably, some lncRNAs in Exos are involved in the therapeutic mechanisms of BMSCs in cardiovascular diseases [13, 14, 45–47]. In our study, lncRNA GAS5, a widely known tumour suppressor that participates in myocardial injury, was proved to be upregulated in BMSC-Exos in this study, which is coincident with previous claims [48]. Wu et al. [26] reported that silencing of GAS5 ameliorated myocardial IR injury in hypoxia/reoxygenation-treated cardiomyocytes and IR rats [26]. Li et al. [49] revealed that silencing GAS5 inhibits inflammation and apoptosis in hypoxia-treated cardiomyocytes [49]. Moreover, GAS5 can mediate oxidative stress in cardiac vascular injury. For example, Diao et al. demonstrate overexpression of GAS5 and its inhibited role in ROS and SOD activity in cardiac microvascular endothelial cells injury model [50]. Xie et al. and Xu et al. also demonstrated the inhibition of GAS5 in oxidative stress in tumours [51, 52]. The above evidences demonstrate that GAS5 is upregulated in BMSC-Exos and may contribute to HF remission, which is in agreement with our results. As expected, we verified the upregulation of lncRNAs GAS5 in BMSC-Exos and revealed that BMSC-Exos carrying GAS5 alleviated HF symptoms in the HF model rats (improved cardiac function and decreased oxidative stress/myocardial apoptosis/pathological damage/fibrosis) and hypoxia-induced injury in the model cells (increased cell viability and decreased G1 phase cells and oxidative stress). Furthermore, GAS5 overexpression enhanced the therapeutic effects of BMSC-Exos in vivo and in vitro. These findings illustrate that exosomal GAS5 derived from BMSCs is beneficial for alleviation in HF.

LncRNAs act as critical regulators of biological processes by regulating gene expression at epigenetic, transcriptional, post-transcriptional, translational, or post-translational levels [53]. Several lncRNA–mRNA regulatory axes are involved in the action mechanisms of MSC-Exos in cardiovascular diseases, such as *KLF3-AS1*/sirtuin 1 (SIRT1) [13], *MALAT1*/autophagy-related 4a (*ATG4a*) [46], urothelial cancer associated 1 (*UCA1*)*-Bcl-2* [54], and *Mir9-3hg/Pum2* [55]. Similarly, many genes, including *CALM2*, *PDCD4*, *TXNIP*, *CYP11B2*, *P2Y12*, and *ROCK1*, have been identified as downstream targets of GAS5 in cardiovascular diseases [17, 18, 23–26]. In this study, UL3 was first identified as the target of GAS5. Our results indicated that exosomal GAS5 may alleviate hypoxia-induced myocardial injury by upregulating UL3 expression. The regulation relationships between GAS5 and UL3 in diseases have not been reported, but the roles of UL3 have been emphasised in previous studies, mainly in several cancers. UL3 is a ribosomal protein that plays an important regulatory role in human cancer. Pecoraro et al. demonstrate that silencing of UL3 abrogates the effects of LQ1 on arresting the cell cycle in the G2/M phase and inducing early apoptosis in colon cancer cells [29]. Furthermore, Russo et al. suggest that the restoration of UL3 re-sensitises lung cancer cells to fluorouracil (5-FU) by inducing oxidative stress [30]. In this study, silencing UL3 weakened the effects of BMSC-Exos in relieving hypoxia-induced injury in vitro, as evidenced by decreased cell viability, increased G1 phase cells, and enhanced oxidative stress, which again emphasise and supplement the pivotal role of UL3 in HF. However, findings regarding the function of UL3 in hypoxia-treated myocardial cells are partially different from those in cancer cells. This difference may be explained by the diverse pathogenesis of different diseases.

The Hippo pathway is an evolutionarily conserved signalling pathway that regulates cellular fate, tissue homeostasis and regeneration, and organ size [56, 57]. The Hippo pathway also plays a crucial role in endogenous heart muscle renewal by regulating cardiomyocyte proliferation, differentiation, and stress responses [58]. Emerging evidence has shown that the Hippo pathway is a therapeutic target for intractable cardiovascular diseases, including HF [58, 59]. In this study, YAP and TAZ, two effectors of the Hippo pathway, were downregulated in HF model rats and cells, which were subsequently upregulated in BMSC-Exos treatment. GAS5 overexpression further enhanced the effects of BMSC-Exos on YAP and TAZ upregulation. These results indicated that BMSC-Exos transporting GAS5 activated the Hippo pathway in HF. In addition, verteporfin, an inhibitor of YAP, weakened the effects of BMSC-Exos in relieving HF in vivo and in vitro. Therefore, we concluded that BMSC-Exos might relieve HF by regulating the GAS5-mediated Hippo pathway.

Ferroptosis is an uncontrolled iron overload condition that can induce oxidative damage in large molecules (DNA, membrane lipids, and proteins) and disrupt the chondrial division and fusion balance [60, 61]. In this study, the action mechanisms of BMSC-Exos related to ferroptosis were analysed. We observed that BMSC-Exos decreased iron deposition and Fe^2+^ levels in HF rats and Fe^2+^ levels in hypoxia-treated myocardial cells. These results indicated an inhibitory role of BMSC-Exos in ferroptosis. Ferroptosis, a critical inducer of programmed cell death, has been identified as a cardioprotective target for HF. For example, Fang et al. (2019) demonstrated that ferrostatin-1 and iron chelation ameliorate IR-induced HF in mice [62]. Furthermore, Chen et al. [34] reported that silencing of TLR4 or NOX4 improves left ventricular remodelling and inhibits cardiomyocyte death in HF rats by inhibiting autophagy and ferroptosis [34]. Therefore, we speculated that BMSC-Exos might relieve HF by inhibiting ferroptosis. This was subsequently confirmed by ferrostatin-1 relieving HF in vivo and in vitro, along with a synergistic effect with BMSC-Exos. Additionally, bioinformatics analysis revealed three GAS5-mediated ceRNA regulatory axes in regulating ferroptosis in HF [63].

Moreover, Hippo pathway has also been demonstrated mediating ferroptosis process through mammalian serine/threonine (Ste20) like kinases 1/2 (MST1/2), large tumour suppressor 1/2 (LATS1/2), and transcriptional coactivator YAP [57, 64, 65]. The studies on regulatory role of Hippo pathway and ferroptosis mainly focus on various cancers. For example, in renal cell carcinoma, TAZ regulates expression of EMP1 and thus induces the expression of a renal-enriched ROS-generating enzyme essential for ferroptosis [66]. In prostate cancer, CYLD regulated ferroptosis through Hippo/YAP signalling [67]. However, the regulation of Hippo in ferroptosis in HF has not been emphasised. In this study, the inhibitory effect of BMSC-Exos on ferroptosis was enhanced by GAS5 overexpression but weakened by UL3 silencing or Hippo pathway blocking. These findings illustrate that the GAS5/UL3/Hippo pathway is the mechanism by which BMSC-Exos inhibit ferroptosis.

Nevertheless, the study exhibits limitations. For example, the long-term effects of BMSC-Exos treatment on HF need to be explored in future. Moreover, Exos therapy has been used as active pharmaceutical ingredient or drug carrier in various disease treatments [8, 68]. However, how to extract EXOs with higher purity and ensure the safety of clinical treatment is still a difficult problem, which needs more effort in the future. Even so, this study reveals the relevant mechanisms of BMSC-Exos in alleviating HF, and provides the foundation for the application of BMSCs in the clinical treatment of HF.

## Conclusions

In conclusion, BMSC-Exos relieved HF in model rats and induced hypoxia-induced injury in myocardial cells. The exosomal GAS5-mediated UL3/Hippo pathway contributes to the therapeutic potential of BMSC-Exos in HF by inhibiting ferroptosis. Our findings reveal that BMSC-Exos are a promising therapeutic resource for HF targeting the GAS5/UL3/Hippo pathway. However, the action mechanism of BMSC-Exos in HF is not limited to the GAS5/UL3/Hippo pathway. Further research into more detailed mechanisms is required.

## Data Availability

Data are provided within the manuscript or supplementary information files.

## Abbreviations

Exos: Exosomes
BMSCs: Bone marrow mesenchymal stem cells
HF: Heart failure
lncRNAs: Long non-coding RNAs
HCP5: Histocompatibility leukocyte antigen complex P5
IR: Ischaemia/reperfusion
GAS5: Growth-arrest-specific 5
TLR4: Toll-like receptor 4
NOX4: NADPH oxidase 4
DMEM: Dulbecco’s modified Eagle medium
FBS: Foetal bovine serum
SD: Sprague–Dawley
TAC: Transverse aortic coarctation
LVEDD: Left ventricular end-diastolic diameter
LVESD: End-systolic diameter
EF: Ejection fraction
FS: Fractional shortening
HE: Haematoxylin–eosin
TUNEL: Terminal deoxynucleotidyl transferase dUTP nick end labelling
IHC: Immunohistochemistry
NC: Normal control
qRT-PCR: Quantitative real time-polymerase chain reaction
MDA: Malondialdehyde
ROS: Reactive oxygen species
SOD: Superoxide dismutase
GSH: Glutathione
CCK-8: Cell counting kit-8
RIP: RNA immunoprecipitation
FISH: Fluorescence in situ hybridisation
NEAT1: Nuclear paraspeckle assembly transcript 1
MALAT1: Metastasis-associated lung carcinoma transcript 1
SIRT1: Sirtuin 1
ATG4a: Autophagy-related 4a
UCA1: Urothelial cancer associated 1
